# Multifunctional aspects of *Piriformospora indica* in plant endosymbiosis

**DOI:** 10.1080/21501203.2019.1600063

**Published:** 2019-03-31

**Authors:** Jisha S, Sabu Kk, Manjula S

**Affiliations:** a Biotechnology and Bioinformatics Division, Jawaharlal Nehru Tropical Botanic Garden and Research Institute, Thiruvananthapuram, India; b Division of Plant Molecular Biology, Rajiv Gandhi Centre for Biotechnology, Thiruvananthapuram, Kerala, India

**Keywords:** *Piriformospora indica*, endosymbiosis, *Cucumis sativus*, phytohormones

## Abstract

(Hymenomycetes, Basidiomycota) is an endophytic fungus that colonises plant roots, and was originally isolated from Rajasthan desert. It is comparable to Arbuscular Mycorrhizal (AM) fungi in terms of plant growth promotional effects. *P. indica* has been used as an ideal example to analyse the mechanisms of mutualistic symbiosis. Major benefit of *P. indica* over AM fungi is that it is axenically cultivable in different synthetic and complex media. A preliminary attempt was made to scrutinise the role of *P. indica* co-cultivation on seedling vigour of common vegetables like *Cucumis sativus* L., *Abelmoschus esculentus* (L.) Moench, *Solanum melongena* L. and *Capsicum annuum* L. The positive effect of *P. indica* co-culture on seedling performance was compared to the effects of growth hormones like indole acetic acid and benzyl amino purine when supplemented to the MS medium at a concentration of 0.1 mg ml^−1^. An exogenous supply of auxin resulted in enhanced production of roots and cytokinin supplement favoured shoot production, whereas *P. indica* co-culture favoured simultaneous production of shoot and root over the control. *P. indica* colonisation inside the roots of *C. sativus* L. was also successfully established. These preliminary results indicate the prospective role of *P. indica* in vegetable farming through its favourable effect on plant growth.

## Introduction

*Piriformospora indica*, come under Hymenomycetes, Basidiomycota (Varma et al. 2001; Weiss et al. 2004) is a growth promoting fungus discovered in the Indian Thar desert in 1997 (Verma et al. 1998). Plant endosymbiosis like *P. indica* are categorised by the penetration of living plant cells by a microbial symbiont, followed by a period during which the symbiont lives partially or completely inside plant cells (Parniske 2000). In contrast to AM fungi, *P. indica* can be easily cultivated on several defined synthetic media and it enhances plant biomass growth in a broad spectrum of plants including Angiosperms, Gymnosperms, Bryophytes and Ferns (Pham et al. 2004). *P. indica* has been used in broad range of plants to provide enhanced nutrient uptake, resistance to pathogens, enhanced secondary metabolites, biomass growth to a variety of plants. This fungus has been used as a model to study the mechanisms and evolution of mutualistic symbiosis (Jacobs et al. 2011; Nongbri et al. 2012). Cell Wall Extract (CWE) is the active fraction from the liquid culture of *P. indica* and it has proven elicitor properties, which was evidenced by our earlier work conducted in *Centella asiatica* (Jisha et al. 2018a). The presence of *P. indica* also had protective role in alleviating stress (Jisha et al. 2018b). CWE is reported to be with fungal exudates and other primary metabolites which can enhance the biomass growth in plants (Verma et al. 1998). Vadassery et al. (2009) reported that these active constituents of the endophytic fungus *P. indica* also stimulate enhanced growth and seed production in *Arabidopsis thaliana*. This heat-stable fraction is able to stimulate root and shoot growth. Cellotriose, a novel chemical mediator, was found to help the complex *P. indica*–plant mutual relationship in symbiotic associations (Johnson et al. 2011).

**Figure 5.**
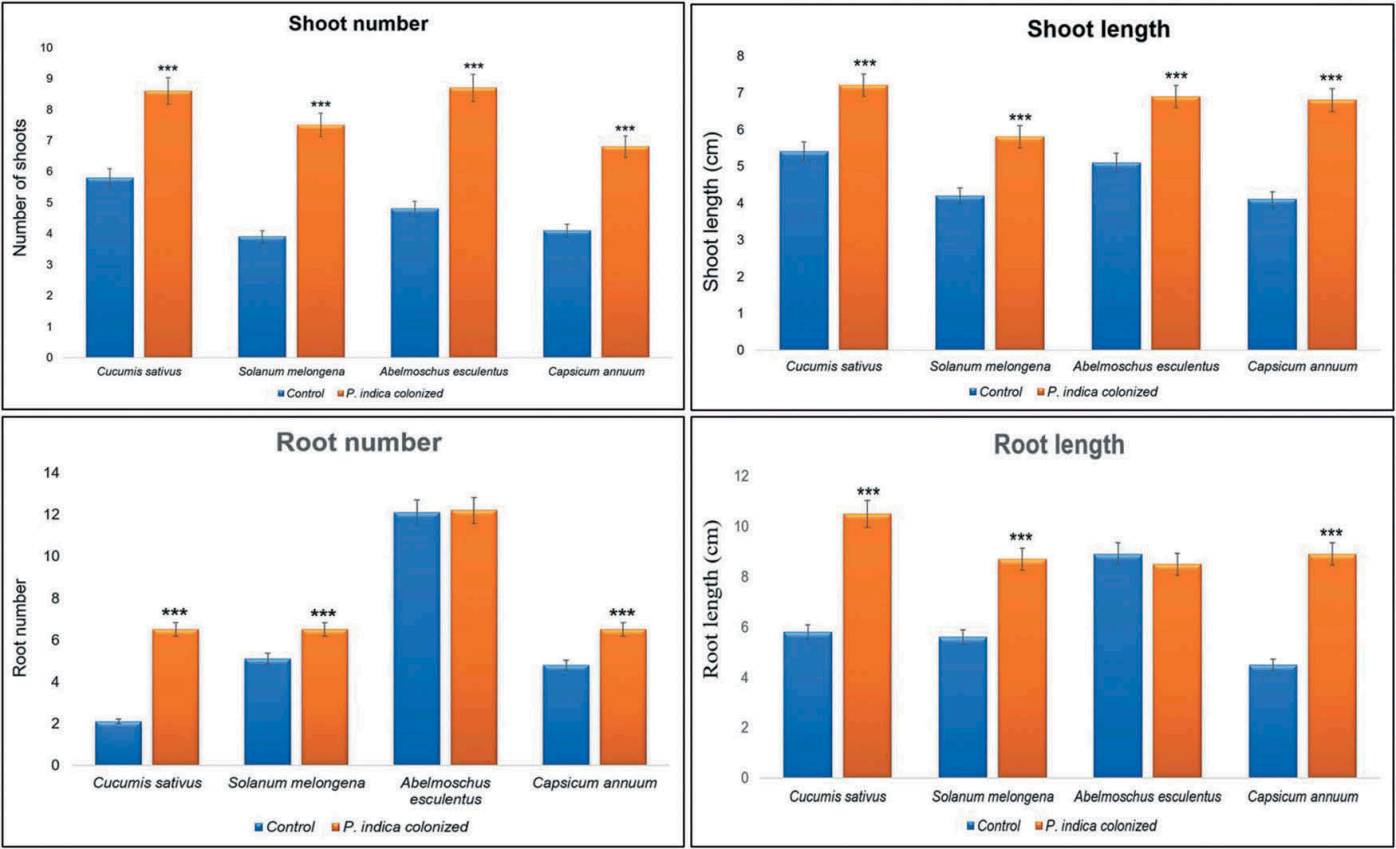
Comparative analysis of phenotypic traits in control and *P. indica* co-cultured plants. Phenotypic traits like shoot number, shoot length, root number and root lengths of control and *P. indica* co-cultured *Cucumis sativus* L., *Abelmoschus esculentus* (L.) Moench, *Solanum melongena* L. and *Capsicum annuum* L. were measured after a period of 1 month. *** indicates *P* < 0.001.

*P. indica* is able to transfer growth-promoting activity to mono- and dicotyledonous plants (Verma et al. 1998; Pham et al. 2004; Barazani et al. 2005 and Jisha *et al*, 2011). Hosts include the cereal crops such as rice, wheat, barley as well as many Dicotyledoneae, including *A. thaliana*. In spring barley, *P. indica* colonisation enhanced plant biomass which was accompanied by grain yield increases of up to 11%. *P. indica* stimulates adventitious root formation in ornamental cuttings (Pham et al. 2004), while enhanced salt tolerance has been observed in barley (Waller et al. 2005). *P. indica* is reported to increase the drought tolerance in *Arabidopsis* (Sherameti et al. 2008) and *Hordeum vulgare* (Waller et al. 2005). *P. indica* enhanced the antioxidant activities in order to cope up with the stress generated in the plants (Vadassery et al. 2009).

The present study was aimed to realise the role of *P. indica* in the germination and vigour of seedlings *in vitro*. The study was conducted in *Cucumis sativus* L., *Abelmoschus esculentus* (L.) Moench, *Solanum melongena* and *Capsicum annuum* In addition, a comparative analysis between the growth hormones and *P. indica* in the biomass growth of *C*. *sativus* was also carried out. The rapid and fast seed germination could be due to the higher rate of water absorption from the media. This is the first report on rapid seed germination in these vegetables and enhancement in plant biomass in *C. sativus* in response to the presence of *P. indica*.

## Methodology

### *Optimisation of* P. indica *growth*

**Figure 1.**
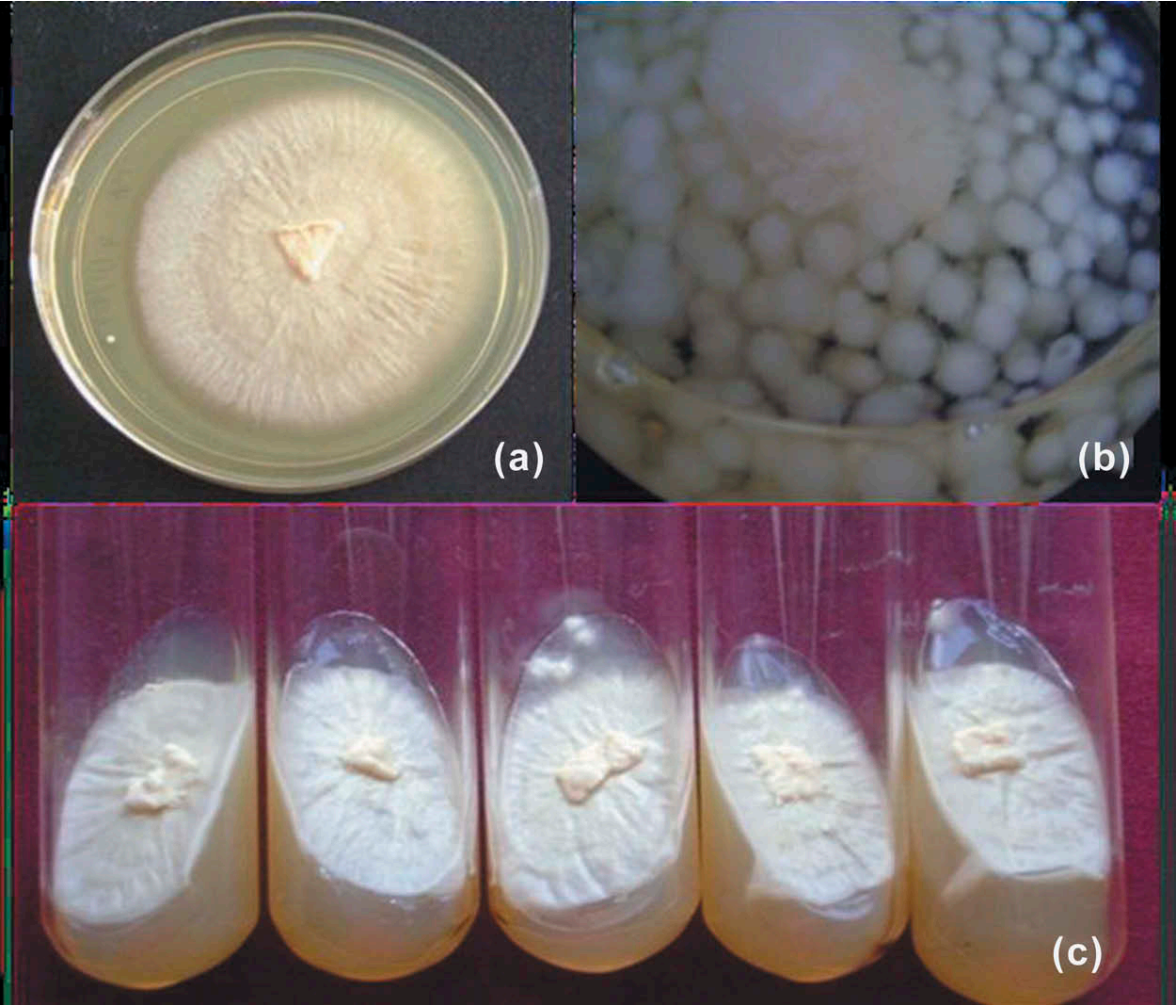
Maintenance of *P. indica* in potato dextrose containing media. (a,c) – *P. indica* maintained in PDA and (b) – *P. indica* maintained in PDB.

Experiments were conducted for optimising the best medium for growth and maintenance of *P. indica in vitro*. Potato dextrose broth (PDB) and Potato dextrose agar (PDA) showed the best and maximum growth (Figure 1). Subculture was performed with the mycelia discs once in a month to maintain the fungal viability to maximum. *P. indica* appeared mat like with several concentric rings in PDA (Figure 1(a,c)) and as globular balls in PDB (Figure1(b)).

### *Surface sterilisation and co-cultivation of seeds with* P. indica

Seeds of *S. melongena* L., *A. esculentu*s and *C*. *annuum* were soaked in 1% detergent solution for about 1 h and washed thoroughly under running tap water. Surface sterilisation was done with 0.01% HgCl_2_ for 8 min followed by a final rinse (three to four times) with sterile double distilled water. The seeds were transferred to medium containing MS (Murashige and Skoog 1962) and PDB (containing *P. indica*) in a 1: 1 ratio and incubated in 16 h: 8 h light/dark at 23 ± 2°C and 55–65% humidity and a light intensity of 25 μmol m^−2^ s ^−1^ provided by white fluorescent tubes, for a period of 30–45 days in the Plant Tissue Culture Laboratory at Jawaharlal Nehru Tropical Botanic Garden and Research Institute, Thiruvananthapuram, India. Normal MS medium devoid of *P. indica* was used as control. Surface sterilised seeds were inoculated in control and *P. indica* containing MS medium for comparison. After 45 days of co-culture, root colonisation was assessed as percentage colonisation (Giovanetti and Mosse, 1980). Three Petri plates each with three seeds of *S*. *melongena* L., five seeds of *A*. *esculentu*s, seven seeds of *C*. *annuum* and four seeds each of *C*. *sativus* L. were analysed for fast seed germination and phenotypic traits.

### P. indica *co-cultivation with* C. sativus *L.*

Seeds of *C*. *sativus* L. were also surface-sterilised by the method described above. The seeds were also inoculated to the media containing cytokinin (0.1 mg l^−1^ benzyl amino purine; BAP) and auxin (0.1 mg l^−1^ indole acetic acid; IAA) with and without *P. indica* in order to compare the effect of *P. indica* in MS medium.

### Analysis of seed germination and other phenotypic traits

Percentage germination of seeds was analysed after a period of 1 month in all the plants used in the study.

### Statistical analyses

For each experiment, seeds were placed in three Petri plates and each Petri plate was with three seeds of *S*. *melongena* L., five seeds of *A*. *esculentu*s, seven seeds of *C*. *annuum* and four seeds each of *C*. *sativus* L. along with the control non-colonised treatments. Analysis of data was carried out using the Graphpad Instat version 3.6 (Graphpad Software Inc., La Jolla, CA, USA). For analysis of growth parameters, in each experiment, six control plants and six *P*. *indica*-colonised plants were analysed and the experiments were repeated twice.

## Results

### *Beneficial role of* P. indica *in seed germination*

**Figure 2.**
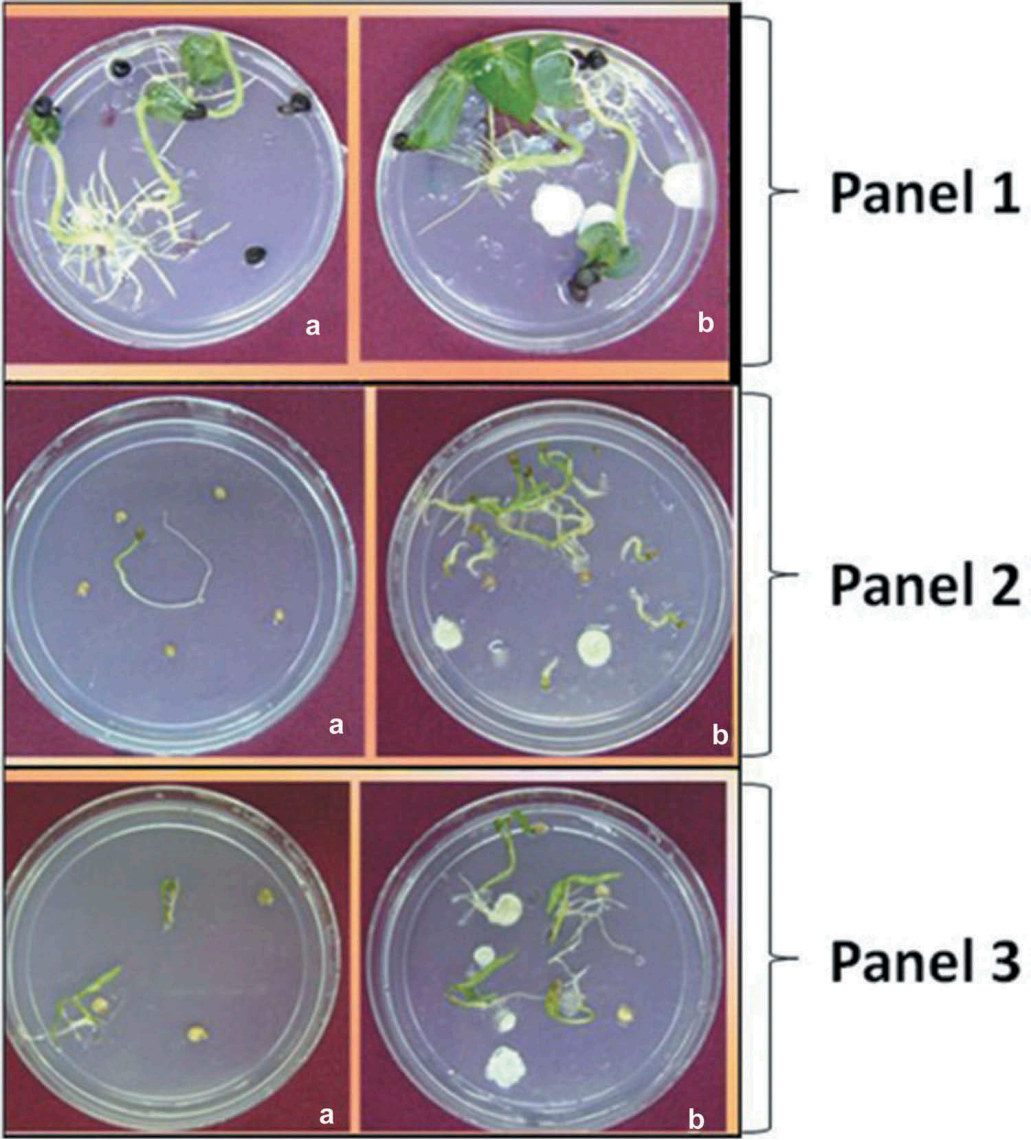
Effect of *P. indica* co-cultivation in the seeds of different vegetables. Vegetables include *Abelmoschus esculentus* (L.) Moench (panel 1); *Solanum melongena* L. (panel 2) and *Capsicum annuum* L. (panel 3). (a) – control seeds; (b) – *P. indica* co-cultured seeds. Number of replications (*n*) = 3.

In this experiment, enhanced vigour was observed in seeds grown in the presence of *P. indica* over the control (Figure 2). Performance of these plant seedlings in terms of germination rate and vigour was strongly promoted by *P. indica* under *in vitro* conditions. Consistent results were observed in all technical and biological replicates (*n* = 3). It was noted that *P. indica* strongly interacted with the roots of these plants resulting in efficient colonisation. Hyphae and spores were detected around the roots and root hair, in the extracellular space and within root cells. The growth promoting effect was visible after 30 days in culture of *A*. *esculentus* (L.) Moench (Figure 2 Panel 1), *S*. *melongena* L. (Figure 2 Panel 2) and *C*. *annuum* L. (Figure 2 Panel 3).

### P. indica *co-cultivation with* C. sativus *L.*

**Figure 3.**
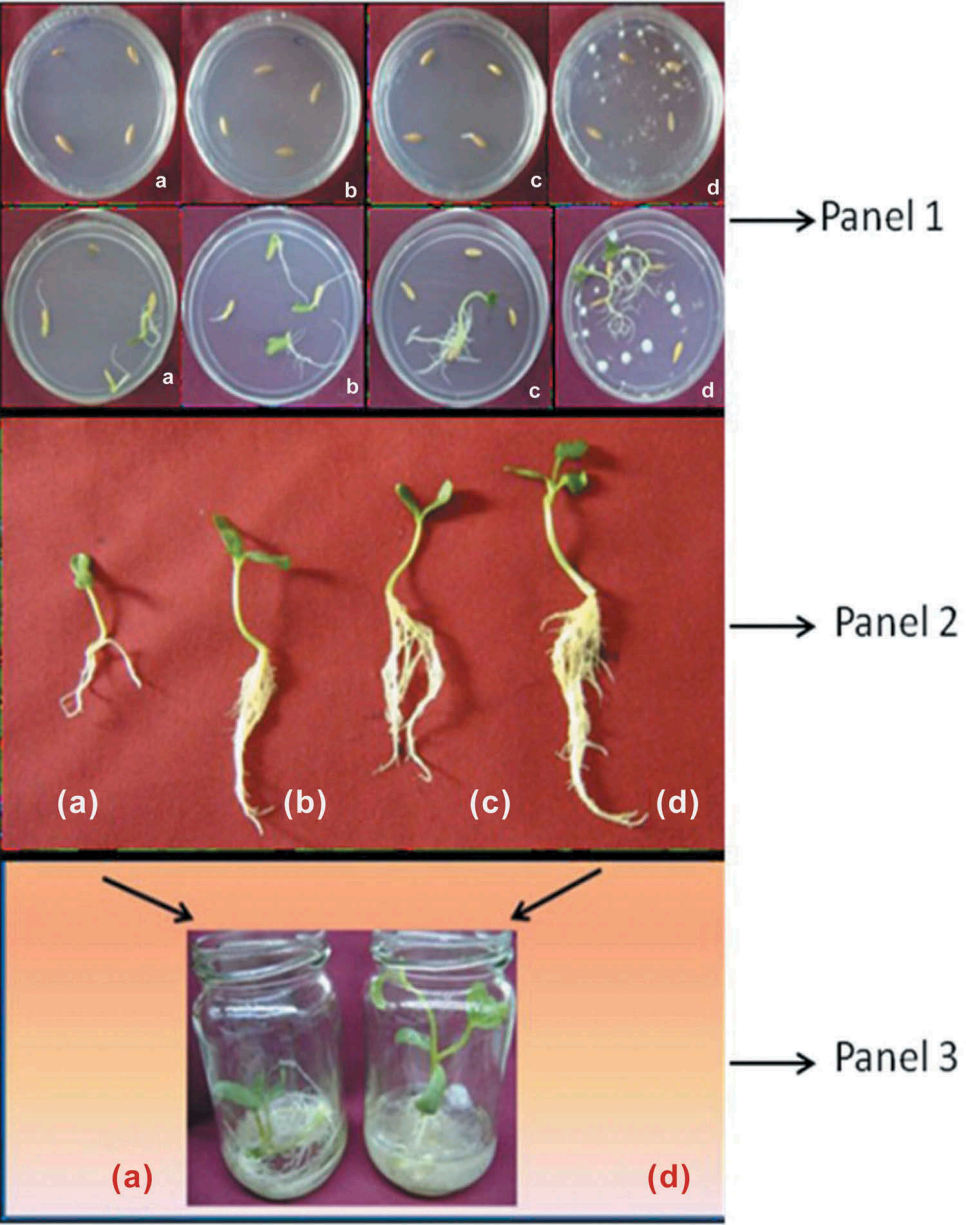
Effect of *P. indica* co-cultivation in *Cucumis sativus*. L. Panel 1: (a) – control seeds (in MS–PDA media in the ratio 1:1); (b) – seeds in cytokinin, BAP (0.1 mg l-1) containing medium; (c) – seeds in auxin, IAA (0.1 mg l-1) containing medium and (d) – seeds in *P. indica* co-cultured medium. Number of replications (*n*) = 3. Panel 2: (a) – Control *C. sativus* (in MS–PDA media in the ratio 1:1); (b) – *C. sativus* in cytokinin, BAP (0.1 mg l-1) containing medium; (c) – *C. sativus* in auxin, IAA (0.1 mg l-1) containing medium and (d) – *C. sativus* in *P. indica* co-cultured medium after a period of 2 weeks. Panel 3: (a) – control *C. sativus* (in MS–PDA media in the ratio 1:1) and (d) – *C. sativus* in *P. indica* co-cultured medium after a period of 2 weeks.

An exogenous supply of auxin resulted in enhanced production of roots, and cytokinin supplement favoured shoot production (Figure 3(b)), whereas *P. indica* co-culture favoured simultaneous production of shoot and root (Figure 3(d)) over the control. *P. indica* colonisation inside the roots of *C*. *sativus* L. is also successfully established. These preliminary results indicate the prospective role of *P. indica* in vegetable farming through its favourable effect on plant growth. In *C*. *sativus*, root and shoot lengths as well as number of root and leaves showed a marked increase in *P. indica*-challenged plants compared to control. A correlated increase in chlorophyll content was also observed indicative of possible increase in photosynthetic efficiency. In all cases, the growth enhancement effected by *P. indica* colonisation in *C. sativus* was more pronounced compared with auxin and cytokinin treatments (Figure 3).

### Comparative analysis of seed germination and phenotypic traits

**Figure 4.**
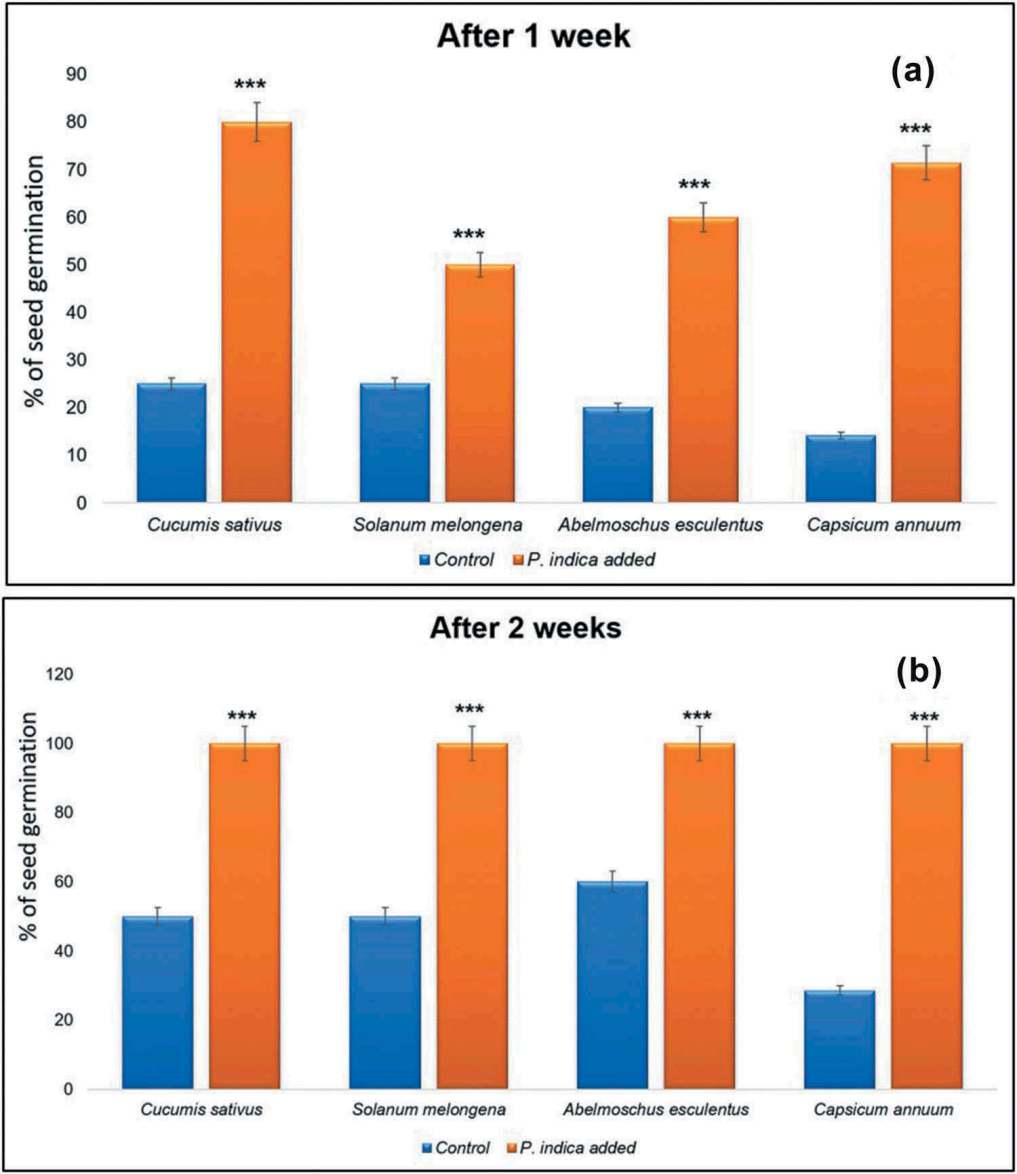
Comparative analysis of seed germination percentage in different plants. Seed germination percentage was analysed in *Cucumis sativus* L., *Solanum melongena* L., *Abelmoschus esculentus* (L.) Moench and *Capsicum annuum* L. after a period of (a) 1 week and (b) 2 weeks. *** indicates *P* < 0.001.

Germination rate was calculated as percentage seed germination after 1 (Figure 4(a)) and 2 weeks (Figure 4(b)) interval. Seeds grown in *P. indica* added media showed fast seed germination rate in comparison with the normal *in vitro* grown plants. Early seed germination and fast growth were pronounced (*P* < 0.001) even after a period of 1 week. Hundred percentage germination was observed in *P. indica* added plants after a period of 2 weeks. Comparative analysis of phenotypic traits like shoot number, shoot length, root number and root lengths were noted after a period of 1 month in all the plants used in the study. *P*. *indica*-colonised *C*. *sativus* L., *S*. *melongena* L., *A*. *esculentus* (L.) Moench and *C*. *annuum* L. showed significant enhancement (*P* < 0.001) in shoot number, shoot length and root lengths. The enhancement in root number was observed in *C*. *sativus* (L.), *S*. *melongena (*L.), and *C*. *annuum* (L.), *whereas* *A*. *esculentus* (L.) Moench did not show any improvement in number of roots (Figure 5).

## Discussion

Different growth promoting microorganisms were discovered recently to cope up with the augmenting requirement for soil nutrients. Agaricomycetes fungus, *P. indica*, can be used as a suitable alternative for enhancing soil fertility. The growth promotion achieved by *P. indica* decreases the fertiliser requirement in soil which diminishes the risk of over application of fertiliser and resulting fertiliser contamination in the environment. *P. indica*-induced seed germination and stimulation was earlier observed in orchids (Blechert et al. 1999) and it also helps to promote seed yield and quality of *Brassica napus* (Zen-zhuSu *et al*, 2017). Varma et al. (2014) reported that *P. indica* culture filtrate promotes plant growth and seed germination in *Helianthus annuus* and *Phaseolus vulgaris*.

Co-cultivation of *P. indica* with seeds of common vegetables indicated that co-cultured seedlings were superior in growth leading to early seed germination and fast growth. The enhanced water absorption in the presence of *P. indica* could the reason behind the fast generation of seedlings. It is also observed that the roots were heavily colonised and produced a large number of chlamydospores observed under *in vitro* conditions. This observation opens scope for application of the plant-promoting symbiotic fungus *P. indica* for better production of crops of agricultural and horticultural importance.

The present study in *C*. *sativus* also confirmed the potential of *P. indica* to be an alternative for the growth hormones like auxins and cytokinins. It was also reported earlier that *P. indica* has the potential to synthesise auxin IAA (Sirrenberg et al. 2007) and the study also explores the role of phytohormones, auxin (IAA) and cytokinins (BAP) in the interaction between *C. sativus* and *P. indica*. The endogenous auxin, IAA, levels were higher in colonised roots compared with the non-colonised controls which points out the hormone dependent growth of *C. sativus* on co-cultivation with *P. indica*. The same pattern of result was also observed with the plant beneficial fungus such as *Trichoderma virens* which enhances biomass production through an auxin dependent mechanism in *A*. *thaliana* (Contreras-Cornejo et al. 2009). Cytokinins act in concert with auxin. These two are balancing each other having generally opposite effects (Campbell et al. 2008). The role of cytokinin, trans-zeatin in the mutualistic interaction between *Arabidopsis* and *P. indica* was reported earlier. In comparison with auxin, high levels of cytokinins were present in colonised roots compared with the uncolonised controls in *Arabidopsis*. These studies show potential of *P. indica* to be a new substitute to plant hormone application. Findings of the *in planta* studies indicate the feasibility of this symbiotic association as a reliable model for further studies on detailed molecular and physiological mechanisms involved in symbiotic association and enhancement of plant secondary metabolite production.

Along with the application of *P. indica* as a plant promoter in a broad range of plants, it is also used as biofertiliser, bioregulator, bio-herbicide, immunomodulator, phytoremediator, biocontrol against insects and pathogens, biotic and abiotic stress tolerance antioxidative agent and biohardening agent (Varma et al. 2014). *P. indica* is capable as a biohardening agent in different *in vitro* plants tested (Sahay and Varma 1999). *P. indica* also enhances early flowering in *Arabidopsis* through photoperiod and gibberellin pathways (Pan et al. 2017). Although the fungus was isolated from hot conditions, *P. indica* has the ability to withstand both the extreme cold and hot conditions. The cold tolerance was evidenced by the experiment in the cold deserts of Leh-Ladakh (Varma et al. 2014). Recently, *P. indica* is documented to reduce the effects of heavy metal stress and DNA impairment during seed germination (Nanda and Agrawal 2018). Thus *P. indica* is unique with its multifunctional effects. The potential of this fungus in plant growth enhancement is yet to be exploited commercially. For augmenting economic and medicinal productivity in plants, we commonly rely on chemical fertilisers. There are many disadvantages of using chemical fertilisers, which accumulate in the soil, causing long-term imbalances in soil pH and fertility.

## *P. indica* – the future prospective

*P. indica* receives pronounced attention in the current scenario, due to its multifunctional properties in the field of agriculture. To work in flow with nature, identification of the active component from *P. indica* which is responsible for the stimulatory effects is of great importance. The biostimulant from *P. indica* can be thus a proper alternative for the chemical fertilisers. Recently, the symbiosis-related metabolites were identified in the non-colonised and *P*. *indica*-colonised Chinese cabbage roots which confer its beneficial role (Hua et al. 2017). A future study involving the isolation and characterisation of a biostimulant from *P. indica* will help in agricultural advancement, as *P. indica* is documented with efficient biocontrol and biofertiliser effects (Varma et al. 2014). The biostimulant from *P. indica* with growth promoting and secondary metabolite augmenting potential can be used as an alternative for a chemical growth promoter. Identification followed by evaluation of the highly potential inducible molecule can be extended to the synthesis of its structural analogues. The development of a *P. indica* “mimic” compound allows the efficient induction of both plant biomass growth and secondary metabolites in medicinally and economically important plants. The compounds with high biological potential can be supplied in standard growth media at normal growth temperatures under various light conditions, which may function through multiple receptors. Future study has also to be extended for the identification of the growth promoting factor from *P. indica* which can be used as a successful biostimulant for plants.
